# Financial Insecurity During the COVID-19 Pandemic: Spillover Effects on Burnout–Disengagement Relationships and Performance of Employees Who Moonlight

**DOI:** 10.3389/fpsyg.2021.610138

**Published:** 2021-02-18

**Authors:** Roziah Mohd Rasdi, Zeinab Zaremohzzabieh, Seyedali Ahrari

**Affiliations:** Department of Professional Development and Continuing Education, Faculty of Educational Studies, Universiti Putra Malaysia, Serdang, Malaysia

**Keywords:** burnout, COVID-19, disengagement, employees’ performance, financial insecurity, moonlight

## Abstract

The novel Coronavirus disease (COVID-19) has magnified the issue of financial insecurity. However, its effect on individual-organizational relations and, consequently, on organizational performance remains understudied. Thus, the purpose of this study was to explore the spillover effect of financial insecurity on the burnout–disengagement relationship during the pandemic. The authors investigate in particular whether the spillover effect influences the performance of moonlighting employees and also explore the mediating effect of disengagement on the relationship between financial insecurity and burnout interaction effect and the performance (i.e., mediated-moderation). This study collected responses from 162 public and private sector employees who are engaged in moonlighting activities in Malaysia. The results from the partial least square structural equation modeling (PLS-SEM) revealed greater levels of financial insecurity and burnout associated with greater levels of work disengagement. The analysis of the interaction-moderation effect showed that when financial insecurity rises, the burnout effect on work disengagement increases among moonlighters. Using the PROCESS macro model, the results displayed burnout as a predictor of extra-role performance via a moderated (financial insecurity) mediation (work disengagement) relationship. Going forward, this study not only opens new avenues for research into the financial consequences of COVID-19 but also calls on managers to take proactive steps to mitigate the negative effect of the pandemic on the performance of moonlighting employees to keep them in the profession.

## Introduction

Financial insecurity refers to the frequency of personal financial concerns and financial stress that interfere with work (Kim and Garman, 2004). An unexpected event, such as COVID-19, may result in these concerns. Due to the COVID-19 pandemic, employees, particularly those engaged in moonlighting (working another job), are generally more aware of the financial security issue. Recent studies on work engagement and job performance have shown that employees ranked financial security as a factor of the highest significance (Kulikowski and Sedlak, 2020). When their employers in mandatory quarantine are unable to provide job protection and income replacement, employees are likely to experience a complicated array of negative emotions and work stress that may impair their work effort and resources. Previous studies on post-coronavirus outbreaks have reported that employees who engaged in any outside employment tend to suffer from enormous financial stress, anxiety, and social isolation that affect their health and productivity (Banerjee and Rai, 2020; Tan et al., 2020).

Furthermore, financial security made vulnerable by COVID-19 could pose drastic functional outcomes for an organization, particularly in the form of increased emotional exhaustion and burnout, impacted disengagement and absenteeism among employees who are double jobholders, and reduced organizational commitment (Russo et al., 2020) and job performance (Sasaki et al., 2020). Hamouche (2020) argued that the pandemic has placed employees at a significantly higher risk of burnout, thus experiencing physical symptoms of stress, such as severe lethargy or exhaustion, and a certain sense of disconnectedness toward work.

However, while a heightened concern is shown toward the financial consequences of the pandemic, less is shown toward the effects it has on moonlighters who fall into a high-risk cohort for burnout and disengagement in organizations. Although there remains an absence of definitive statistics on the prevalence rates of employees’ financial insecurity and burnout during the pandemic, previous studies have found that dual jobholders were impacted by both (Bick et al., 2020; Talaee et al., 2020). Financial insecurity and burnout have been given great attention by researchers and practitioners because of the potential vulnerability on dual jobholders’ well-being and effectiveness (turnover; Betts, 2006(job satisfaction; Sliter and Boyd, 2014; Campion et al., 2020). The major impact of financial insecurity on organizations during the pandemic makes it a key phenomenon that provides opportunities to further investigate how and why it affects organizational functioning.

To adequately address these concerns, this research explores two notable gaps in the extant literature. First of all, we examine the possible relationship between financial insecurity and individual-organizational relations. It is proposed that this relationship transpires from a spillover effect where financial insecurity affects the relationship between burnout and work engagement of employees who moonlight. Specifically, this study tests if these employees’ perceptions of financial insecurity during the pandemic moderates the relationship between burnout and disengagement. Second, the extent of the spillover effect on employee performance is examined. We examine whether the interaction effect between financial insecurity and burnout has implications for the performance of moonlighting employees.

## Hypotheses

### Financial Insecurity, Burnout, and Disengagement

Financial insecurity is employees’ biggest wellness concern in the wake of the coronavirus pandemic. Since the outbreak of COVID-19, many governments have announced an initial movement restriction order (Roberton et al., 2020). The impact of this restriction on an organization would significantly increase the feeling of financial insecurity among employees who moonlight (ILO, 2020). It can harm the mental health of moonlighters who are affected by the reduction of working hours and the organizational reforms of closure. Recently, the National Foundation for Credit Counseling surveyed Americans ages 18 and up, and the results showed that 69% of the respondents’ report experiencing financial insecurity due to the economic fallout as a result of COVID-19 (Consumer Credit, 2020). Researchers have suggested that financial insecurity transpires if one cannot fulfill financial obligations (Odle-Dusseau et al., 2018). According to Hjelm et al. (2017), one of the top contributors to psychosocial stress is financial insecurity since basic living conditions are built upon the management of personal financial resources. Prawitz et al. (2010) showed a significant positive effect of employee financial distress on high absenteeism. Sinclair and Cheung (2016) study also found that financial insecurity is negatively associated with work engagement, workers’ performance at the workplace, and organizational commitment. Given this, financial insecurity and other personal finance-related stress can result in negative outcomes among employees at work.

According to the Conservation of Resources (COR) theory, individuals strive to reduce the depletion of resources (Hobfoll, 2011), and to overcome this, they may seek additional employment to replace any loss of resources (Park et al., 2014). A study examined the extension of this experience and found that when the substitution of diminished resources is not possible, the individuals may simply disconnect from the situation as a means to reduce further losses (Whitman et al., 2014). Therefore, it can be rationalized that if financial security is considered a resource, then what threatens it is financial insecurity. Furthermore, as far as work disengagement is concerned, the fear of losing an income during the pandemic itself may outweigh the actual income loss. Given the perceived threat to their finances, it is likely that employees begin to disengage from work and seek alternative employment. Thus, we proposed that:

**Hypothesis 1**: *There is a positive relationship between financial insecurity and disengagement.*

There is no doubt that the COVID-19 situation has created ambiguities and uncertainties for organizations to function. This has required organizations to step-up in safeguarding employee wellness. By extending the Job Demands-Resources (JD-R) model (Bakker and Demerouti, 2017) in the COVID-19 situation, it can be observed that across and within industries, there exists a variety of effects on both job demands and resources. Present evidence suggests a deterioration of working conditions in general, and this may apply to moonlighters as well. In light of these constraints, COVID-19 has substantially increased the prevalence of job burnout encounters, which bring forth a chronic stress syndrome that encompasses chronic exhaustion and feelings of disconnect with work (Demerouti et al., 2017). Furthermore, previous studies have found that job demands lead to an increase in emotional exhaustion while job resources (or lack thereof) result in work disengagement (Peterson et al., 2008; Kaiser et al., 2020).

**Hypothesis 2:** *Job burnout will have a positive relationship with disengagement.*

It is noteworthy to mention that the relationship between burnout and disengagement has been recognized in the JD-R model (Bakker et al., 2008). According to the JD-R theory, financial insecurity can be considered a demand that exacerbates health impairment conditions contributed to by the exhaustion of mental and physical resources. This continuing state of depletion has been connected with the employee’s state of disengagement; the onset of negative work attitudes begins to develop as demonstrated by a distant sense between self and work (Demerouti et al., 2017). According to the JD-R theory, demands are burnout predictors, and lack of resources is the main antecedent for less than desired engagement levels. As such, we put forth the argument that financial insecurity can attenuate engagement because the twin effect of demands and lack of resources may result in employees’ work goals being unmet and their growth and development being hindered. From the employer, the employee expects job safety and security, but the lack thereof converts into the threat of financial insecurity. This demand serves as a signal for employees to adjust their willingness to perform; when their employer does not appreciate their loyalty and dedication, they perform at a less than desired work engagement quality (Parzefall and Hakanen, 2010). The extant empirical research has provided evidence on the negative effects of financial insecurity on work engagement. However, the aspect and effect of financial resources in Kahn (1990) grounded findings remain ambiguous. Therefore, this study considers financial insecurity as the likely moderator of the association between burnout and disengagement. We further extend this approach by converging it with Baron and Kenny (1986) moderation approach, whereby the moderator variable can affect a change—of direction and/or strength—in the relationship between an endogenous and an exogenous variable. We contend that the strength of an individual’s financial insecurity is likely to intensify the interaction of burnout toward work disengagement. However, it must be noted that earlier studies have realized a weak relationship between these two variables (Basinska and Gruszczynska, 2020). Researchers argued that this can be deducted as an employee’s rational response. An employee copes with financial insecurity by exerting effort while maintaining job performance to assert him/herself as a valuable asset to the organization (Wang et al., 2015). Despite this argument, other researchers alluded that employees’ behaviors and reactions in the incidence of financial instability are not limited to individual factors, they are also reliant on the organization’s treatment toward their employees (Lin et al., 2018). Price et al. (2002) found that employees who suffer from economic stress are those who are faced with strikingly challenging outcomes that push beyond physical and mental health conditions, diminish life satisfaction, and intensify the probability of emotional exhaustion and burnout. It must be noted that while several studies have connected financial insecurity perception to reduced organizational commitment and higher levels of disengagement (Lambert et al., 2010), the general perception is that when the financial insecurity stressor increases, burnout levels will increase, and work engagement levels will decrease. Thus, we proposed that:

**Hypothesis 3**: *Financial insecurity moderates the relationship between burnout and disengagement, so that when financial insecurity is elevated during the pandemic, the relationship will be stronger than when financial insecurity is low.*

### The Mediating Role of Work Disengagement

Work disengagement relates to work engagement; numerous studies have examined the interaction between the two, where disengagement is discussed as a negative influence on the organization (Rastogi et al., 2018). Kahn (1990) considered the following concept of personal disengagement that describes the issue of disengagement, which leads to the decoupling of the self from job role. He defined personal disengagement as the simultaneous defense and withdrawal of an individual’s preferred self in behaviors that encourage a lack of connection, physical, cognitive, and emotional absence, and incomplete role performance.

In a scenario where financial resources are denied, an employee’s sense of being able to achieve any goal can be dampened, which leads to inherent failures, frustrations, and disengagement (Bakker et al., 2008), thus leaving them vulnerable to stress. The COR framework provides that individuals tend to avoid the depletion of resources. Performing their duties to the extent of their ability or in compliance with the organizational expectations can be untenable for those who are disengaged (Wang et al., 2015). This is because doing so is likely to cause additional resource losses. Accordingly, the COR model predicts that employees with fewer resources as well as employees with initial resource losses might face future resource losses (Halbesleben et al., 2014). Thus, starved for financial resources, disengaged employees would seek to minimize discretionary outputs and also reduce efforts in their actual job, reducing employees’ in-role and extra-role performance (Bakker et al., 2008).

In particular, moonlighting employees would be disengaged as a consequence of the lack of financial resources combined with the failure of personal and social support to compensate for the loss. Therefore, seeking to avoid any further loss of resources, they could opt to restrict their performance. However, disengaged employees are likely to exhibit a lack of commitment combined with leaving their jobs. This is based on the model of COR. Denied job resources, disengaged employees are highly reluctant to expand resources in being committed to the job or the organization. This may manifest into the reduction of the affective commitment and the increase in the turnover intention (Mohd Rasdi and Tangaraja, 2020). Shuck et al. (2011) argued that turnover is the final act of disengagement. Thanacoody et al. (2014) further extend disengagement as a key mediator between exhaustion and turnover intention. Burnout affects work performances above and beyond disengagement (Rožman et al., 2018). Based on the COR framework, we, therefore, assume the interaction effect of burnout and financial insecurity on work disengagement to reduce moonlighting employees’ in-role and extra-role performances.

These results strengthen our conviction that disengagement should affect the burnout-employee performance relationship. To this effect, it is speculated that the conditional relation between burnout (reliant on the various levels of financial insecurity) and performance may occur primarily via its association with the disengagement variable. The hypotheses described above illustrate a mediated-moderation that arises when the relationship between the independent variable and variable Z affects the mediator variable, which eventually impacts the dependent variable (Preacher et al., 2007). Thus, the propositions are as follows:

**Hypothesis 4**. *Work disengagement mediates the relationship between burnout × financial insecurity interaction and in-role performance*.

**Hypothesis 5**. *Work disengagement mediates the relationship between burnout × financial insecurity interaction and extra-role performance.*

## Materials and Methods

### Research Design

A cross-sectional survey design was employed in this study. A cover letter was provided to explain that the sole intention of the survey was for academic research to extend the understanding of how financial insecurity interacts with job stress, and this could be a concern for organizations operating in the COVID-19 era.

### Participants and Procedures

Using the cross-sectional technique, the researchers conducted data collection in April and May, in 2020. The target population is defined as private and public sector employees who are dual jobholders. The inclusion criteria for respondents were those who: (a) moonlight as ride-hailers, (b) ride-hail after the working hours of their primary job, and (c) stay in Malaysia’s Klang Valley metropolitan area. In a PLS-SEM context, researchers recommended using G^∗^Power (Hair et al., 2017) and as such, this study employed G^∗^Power Version 3.1. According to Faul et al. (2009), the sample size should be 150 to serve small effect sizes. With this, G^∗^Power can effectively analyze the probabilities of significant associations when examining numerous latent variables invalidated relationships (Baroudi and Orlikowski, 1988). As per Cohen (1988), power is set at a value of 0.80. In this study, 0.99 is the estimated power for the base model which was beyond the 0.80 cut-off value. Therefore, the power greater than 0.80 confirmed that sufficient confidence was attained on the hypothesized relationships of this study. It must be noted that this study had abided by the ethical guidelines of the American Psychological Association. Besides, UPM Ethics Committee for Research Involving Human Subject approved the study protocol as registered as JKEUPM-2020-180.

The study used the systematic sampling method as a simple random method of sampling that applies a constant interval to choosing a sample of elements from the sampling frame (Chauvet, 2020). Approximately 100,000 ride-hailers working in three major companies (e.g., Grab, MyCar, Gojo) in Malaysia, and due to no minimum hours required to drive, more than half of them are moonlighting and working in public and private companies in Malaysia. After obtaining the list and emails of drivers who moonlights from the companies, we used a random number generator available online to select the moonlighters. Then, the approved questionnaire was distributed electronically to participants. The survey was open to online responses for 8 weeks and to encourage participation, e-mail reminders were sent at 1, 3, and 5 weeks after the initial invitation and before survey closure. Of the 180 questionnaires distributed, 162 complete questionnaires were returned (90% return rate).

### Measures and Variables

#### Exogenous Variables

##### Financial insecurity

This study employed Munyon (2008) ten-item instrument to assess financial insecurity, which reveals an employee’s level of certainty regarding his or her future financial security. This scale has a Cronbach alpha of 0.77. The sample items include: “I believe that I have enough savings for an emergency” and “I have financial stability.” Responses ranged from not at all (1) to completely agree (6). For each item in the scale, reverse coding was conducted where higher scores represent higher perception levels of financial insecurity.

##### Job burnout

Maslach and Jackson (1981) eleven-item burnout scale was employed to measure the state of psychological, emotional, and physical stress that manifests from extended exposure to occupational stress. This scale consists of a feeling of emotional exhaustion, depersonalization, and reduced professional accomplishment. The sample items include: “I feel emotionally drained from my work” and “I worry that this job is hardening me emotionally.” All of the items were rated on a 5-point Likert scale ranging from never (1) to always (5). Cronbach’s alpha was 0.83.

#### Endogenous Variables

##### Work disengagement

The work disengagement scale was developed in a Chinese version in previous studies (Hui et al., 2012) to measure work disengagement. This scale comprises of work withdrawal and exit-seeking behaviors, and it was used to measure an employee’s self-reported generalized tendencies of the aforementioned behaviors. A sample of items is: “I want to quit the job as soon as possible.” All of the items were rated on a 7-point Likert scale which provided the options range of 0 = *never* to 6 = *always*.

##### Job performance

Job performance was measured from the moonlighter’s perspectives in terms of in-role performance and extra-role performance.

**In-role performance:** It refers to the completion of role and tasks as stipulated in the formal job description, whereas extra-role performance refers to behaviors of a discretionary nature that is not part of the specific job criteria of the employee but also promote effective organizational function (Biswas and Kapil, 2017). The response was rated on a scale of never (1) and always (5). The in-role performance was measured using the 7-item of Task Performance Scale of Goodman and Svyantek (1999). Samples items are: “I complete tasks that are expected of me” and “I adequately complete assigned duties” (*a* = 0.82).

**Extra-role performance:** The 8-item of extra-role performance scale by Eisenberger et al. (2010) was used to measure extra-role performance. A sample item is: “I continue to look for new ways to improve the effectiveness of my work.”

### Data Analysis

The researchers used PLS-SEM to validate the research model developed for this study (Wong, 2013). The authors used the Smart-PLS 3.0 software for data analysis (Ringle et al., 2015) to run the PLS algorithms with a bootstrapping set to 5,000 subsamples (Hair et al., 2016). The PLS method was preferred over other regression models as it can serve the complex study model as well as the small sample size (*n* = 162), thus the suitability of PLS (Hair et al., 2016) as an analysis technique for this research (Carrión et al., 2016).

The interaction-moderation method was used to test the moderating role of financial insecurity in the burnout-work disengagement relationship. The researchers run a bootstrapping procedure to obtain the standard error for *t*-value computation. Confidence intervals that do not include zero have means effects that are significant at α = 0.05. Both the Standardized Root Mean Square Residual (SRMR) and Bentler-Bonett Normed Fit Index (NFI) were used to evaluate model fit. SRMR evaluated the differences between observed and expected correlations whereas NFI presents the incremental measure of goodness of model fit. The rate of missing data for items was less than 3%, and missing data were addressed by the regression imputation method.

### Testing for Moderated Mediation

**FIGURE 1:**
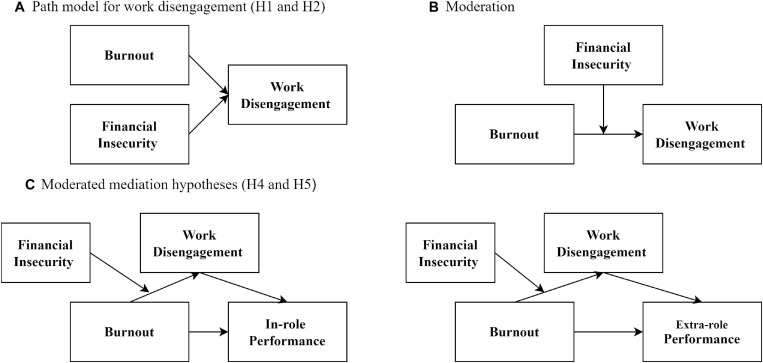
Hypothesized path models. **(A)** For direct effects, **(B)** for moderation, and **(C)** for moderated mediation.

In this study, we determined moderated mediation as to whether a mediational process is conditional on other variables (Hayes, 2017). Going forward, the bootstrap method of Hayes (2012) PROCESS macro was employed to test both direct and indirect effects of X on Y, conditional on a moderator. Given the sheer number of models available through PROCESS, we focused on models involving a single moderator (W). PROCESS model 7 was used to determine the indirect effect of X on Y that varies as a function of W, where W moderates the path from X to M. In hypotheses 4 and 5, we assumed that financial insecurity moderates the association between burnout and work disengagement. As such, we looked into the possibility that financial insecurity may provide a conditional influence on the strength of the indirect relationship between burnout and in-role and extra-role performances. This is demonstrated by a moderated mediation pattern between the variables as shown in Figure 1. We predicted a strong (weak) relationship between burnout and work disengagement when financial insecurity is high (low). In equation form,

**Hypothesis 4:** $\hat{W\,D}$=b_0_+b_1_X_*B0*_+b_2_X_*FI*_+b_3_X_*B0*_X_*FI*_
$\hat{I\,R\,P}$ = b_0_+b_1_X_*BO*_+b_2_X_*WD*_

**Hypothesis 5:**$\hat{W\,D}$=b_0_+b_1_X_*B0*_+b_2_X_*FI*_+b_3_X_*B0*_X_*FI*_
$\hat{I\,R\,P}$ = b_0_+b_1_X_*BO*_+b_2_X_*WD*_

## Results

### Demographic Results

The sample consisted of 116 males and 46 females with an average age of 33.07 years (*SD* = 9.704). The average year they worked as ride-hailers was 2.27 (*SD* = 1.72), while the average income for their primary and secondary jobs was RM2, 674.83, and RM1, 586.48, respectively.

### Measurement Model

The measurement model fulfilled all model fit requirements as per the results shown in Table 1. Firstly, the reliability condition was fulfilled as indicated by factor loadings that scored greater than 0.7. The item-trimming process was used to remove weak loading items in terms of values. As such, items at factor loadings, less than 0.05 were disqualified from the final analysis. Next, Dijkstra-Henseler’s rho indicators were employed to assess construct reliability (Dijkstra and Henseler, 2015). Table 1 shows that the construct reliability of composite indicators was confirmed by the minimum reliability value of 0.7 (Henseler, 2017). Also, the latent variables met the standard requirement of convergent validity because all values of their average variance extracted (AVE) surpassed 0.50 (Hair et al., 2016). Also, the values of Cronbach’s alpha (α) were greater than 0.70 (see Table 1).

**TABLE 1:** PLS-CFA measurement model results.

**Variable**	**Loading**	**M**	***SD***	**α**	**rh-A**	**CR**	**AVE**	**VIF**
In-role performance		4.09	0.705	0.901	0.922	0.922	0.629	2.204
IRP1	0.811							
IRP2	0.835							
IRP3	0.816							
IRP4	0.821							
IRP5	0.81							
IRP6	0.766							
IRP7	0.688							
Extra-role performance		4.08	0.656	0.915	0.922	0.925	0.608	2.336
ERP1	0.83							
ERP2	0.715							
ERP4	0.741							
ERP5	0.867							
ERP6	0.751							
ERP7	0.747							
ERP8	0.745							
Burnout		3.037	0.619	0.849	0.889	0.882	0.52	1.927
BO1	0.651							
BO2	0.646							
BO3	0.797							
BO4	0.899							
BO5	0.703							
BO6	0.658							
BO7	0.656							
Financial Insecurity		2.332	0.619	0.767	0.815	0.847	0.589	1.614
F1	0.825							
F2	0.803							
F3	0.732							
F4	0.685							
Work disengagement		1.832	0.572	0.836	0.866	0.87	0.51	1.88
WD1	0.698							
WD2	0.767							
WD3	0.84							
WD5	0.509							
WD6	0.736							
WD7	0.655							
WD8	0.59							
WD9	0.587							

*Values of composite reliability (CR), average variance extracted (AVE), variance inflation factor (VIF), mean and standard deviation.*

Discriminant validity uses empirical standards to distinguish the degree of one construct to another. Therefore, to conduct discriminant validity, this study applied the Fornell-Larcker and Heterotrait-Monotrait (HTMT) criteria as recommended by Fornell and Larcker (1981) and Henseler et al. (2015). Regarding the Fornell-Larcker criterion, the results indicated that the square root of each construct’s AVE was higher than the correlation values with any other construct. Also, the values of HTMT were below the threshold value of 0.85 in all cases as shown in Table 2. Consequently, this study confirms that burnout, disengagement, financial insecurity, and in-role and extra-role performance could be mutually discriminated in the study.

**TABLE 2:** Measurement model: discriminant validity.

**Fornell-Larcker Criterion**						**Heterotrait-Monotrait ratio (HTMT)**				
	**BO**	**ERP**	**FI**	**IRP**	**WD**	**BO**	**ERP**	**FI**	**IRP**	**WD**
BO	0.721									
ERP	–0.208	0.780				0.252				
FI	0.032	–0.194	0.763			0.172	0.244			
IRP	–0.286	0.710	–0.164	0.793		0.321	0.802	0.216		
WD	0.296	–0.124	0.223	–0.172	0.680	0.289	0.131	0.257	0.178	

*JB, job burnout; FI, financial insecurity; WD, work disengagement; IRP, In-role performance; ERP, extra-role performance.*

### The Measurement Invariance

The researcher conducted the measurement invariance of composite models (MICOM) procedure to establish the measurement invariance requirements. This step ensures that the group-specific model estimations would not be similar stemming from content distinction and the meaning of the latent variables access group (Chin and Dibbern, 2010; Sarstedt and Ringle, 2010; Hair et al., 2017). Known as a systematic assessment of measurement invariance, the MICOM process consists of three steps that evaluate configural invariance, compositional invariance, and equal means and variances, respectively.

In addition to running MICOM and the measurement invariance analysis, a permutation test was also conducted. A nonparametric test, such as the permutation test, serves to observe interchangeably between male and female groups so that a re-estimation of the model can be made for each permutation. To generate the distribution of test statistics, a total of 1,000 repetitions is deemed sufficient. Random cases were assigned amongst male and female groups and the model was estimated, including the test statistics being calculated. The result is considered significant at the 5% probability when the *r*-value is lower than 0.05 or higher than 0.95 for a coefficient difference of a group path (Rigdon et al., 2010). Table 3 displays the results of the invariance measurement testing. The MICOM Step 1 process involved content and expert validity, as well as identical indicators per measurement model across the groups. This also included identical data treatment for missing value treatment and identical algorithm settings. The second step determined if a composite contains a correlation in the male and female groups, respectively, which reported a slight difference thus establishing compositional invariance. Finally, based on the permutation test, Step 3 evaluated the confidence intervals for mean and variance values, all of which indicated the establishment of the full measurement invariance. The permutation tests indicated that all variables (across groups) have effects of significant differences.

**TABLE 3:** Results of invariance measurement testing.

**Variables**	**Compositional Invariance (Correlation = 1)**			**Equal Mean Assessment**				**Equal Variance Assessment**		
	**C = 1**	***CI***	**Partial Measurement Invariance Established**	**Δ**	***CI***	**Equal**	**Δ**	***CI***	**Equal**	**Measurement Invariance**
In-role performance	0.99	[0.88. 1.000]	Yes	0.98	[−0.34,0.35]	Yes	0.04	[−0.48,0.40]	Yes	Full
Extra-role performance	0.88	[0.63, 1.000]	Yes	0.65	[−0.39,0.32]	Yes	−0.06	[−0.72,0.68]	Yes	Full
Burnout	0.98	[0.82,1.000]	Yes	0.83	[−0.31,0.37]	Yes	−0.11	[−0.43,0.46]	Yes	Full
Financial Insecurity	0.99	[0.84,1.000]	Yes	0.95	[−0.36,0.34]	Yes	−0.07	[−0.67,0.60]	Yes	Full
Work Disengagement	0.96	[0.96,1.000]	Yes	0.23	[−0.32,0.35]	Yes	0.10	[−0.75,0.77]	Yes	Full

*CI, Confidence interval; Δ, Differences.*

## Hypothesis Testing

### Structural Model

After the measurement model was validated, we conducted path analysis to test H_1_ and H_2_. The structural model was evaluated through examinations on the significance of path coefficients, effect size (*f*^2^), coefficient of determination (*R*^2^), and predictive relevance (*Q*^2^). The study confirmed that the data fitted the model well because the results of all models show less than 0.08 SRMR values and greater than 0.8 NFI values (Henseler et al., 2016).

**FIGURE 2:**
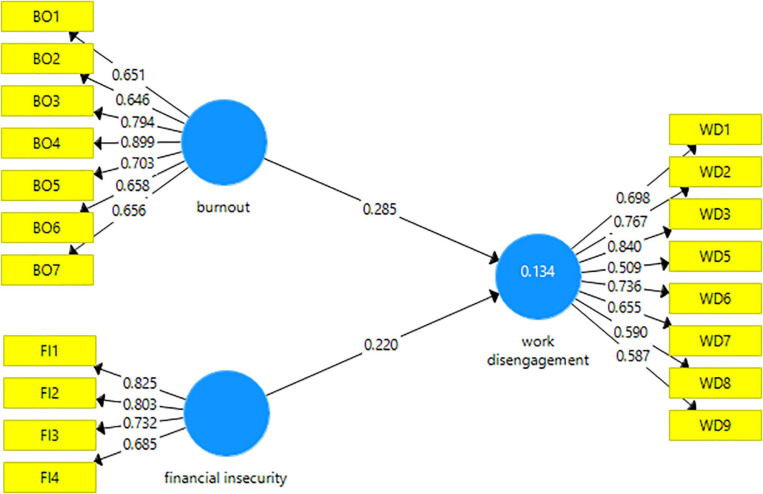
The structural model for work disengagement.

The evaluation of the structural model was conducted using a nonparametric bootstrapping procedure with a resample of 5,000 to generate the β and corresponding *t*-values. The results revealed that burnout significantly explains the work disengagement proportion (β = 0.285, *t* = 3.787, *P*-value = 0.000). Furthermore, the path coefficients show the link between financial insecurity (β = 0.220, *t* = 2.458, *P*-value = 0.014) and work disengagement. As shown in Figure 2, these results fail to reject H_1_ and H_2_. Besides, the calculation of R^2^ estimated the variance value in work disengagement via the explanation of burnout and financial insecurity. Accordingly, if an *R*^2^ value is greater than 0.134, the coefficient of determination is deemed low (Henseler et al., 2015). This study used *f*^2^ to calculate the deletion impact of exogenous variables, namely burnout and financial insecurity, on the endogenous variable of work disengagement. The researchers of this study observed Cohen (1988) *f*^2^ classification as 0.02 = small, 0.15 = medium, and 0.35 = large, respectively. The f^2^ result for financial insecurity was 0.156 while burnout was 0.194, respectively. This showed medium-sized effects in the explanation of work disengagement. This study also used *Q*^2^ for the calculation of predictive relevance job burnout as the endogenous variable. Henseler et al. (2015) *Q*^2^ classifications are 0.02 = small, 0.15 = medium, and 0.35 = large, respectively. The *Q*^2^ result was 0.39, which is an indication of disengagement of work achieving a large predictive relevance.

### Moderating Effect of Financial Insecurity

The results of interaction moderation showed a significant and positive link between burnout and work disengagement (*b* = 0.215, *t* = 2.887, *p* < 0.004). Similarly, a positive and significant relationship was reported between financial insecurity and disengagement of work (*b* = −0.209, *t* = 2.783, *p* < 0.006). Additionally, there was a significant relationship between the burnout-financial insecurity interaction effect and work disengagement (*b* = 0.165, *t* = 2.67, *p* = 0.0490). The moderation test results are reported in Table 4.

**TABLE 4:** Moderation test results.

					**Work Disengagement**		
**Hypothesis**	**β Values**	***T* values**	***P* Values**	***f*^2^**	***R*^2^-without moderation**	***R*^2^-with moderation**	**ΔR^2^-Result**
H_3_: Financial insecurity moderates the burnout-work disengagement relationship.	0.165	2.67	0.0490	0.008	0.134	0.143	0.011 Percent accepted

The interaction chart, as shown in Figure 2, denotes respondents’ level of perceptions of financial insecurity from low to high [±1 standard deviation (*SD*)] where the lower (−1 *SD*), central (mean), and upper (+1 *SD*) lines represent a low, medium, and high level of perceptions of financial insecurity, respectively. When the central line is compared to the upper line, an increase in respondents’ burnout perceptions is associated with a greater rise in disengagement, as their perceptions of financial insecurity are elevated. Additionally, it is observed that the significant differences between slopes indicate that financial insecurity impacted the strength of the burnout-disengagement relationship. Therefore, the results confirmed that the main effect of the moderator on work disengagement has been established in this study, thus the results fail to reject H_3_.

### Moderated Mediating Effect

**FIGURE 3:**
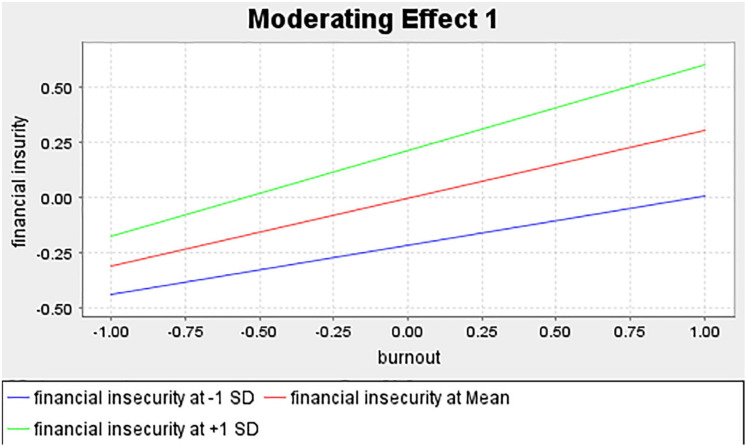
Results of the moderating effect of financial insecurity on the relationship between burnout and disengagement.

According to Hayes (2017), when the moderation relationship is present, in this case between the indirect effect of burnout on in-role and extra-role performances, moderated mediation emerges. This indirect effect corresponds with the value of financial insecurity, which is the moderating variable. To evaluate this conditional process model, this study carried out the index of moderated mediation (Hayes, 2017) where the index quantifies the linear association between the moderator and the indirect effect. Therefore, with the inclusion of zero in the confidence interval (95% CI: −0.247 to 0.738), the moderated mediation hypothesis is not supported, as shown in Tables 5, 6. The indirect effect of burnout on in-role performance through work disengagement does not depend on levels of financial insecurity; the results reject H_4_. In contrast, the results indicated that financial insecurity significantly moderated the indirect effect of burnout on extra-role performance (95% CI: −0.0061 to −0.0021). This study concluded that the index of moderated mediation for extra-role performance is statistically relevant and that H_5_ is failed to reject (see Figure 3).

**TABLE 5:** Results of the bootstrapping analysis.

**Variable**	**Outcome variable: In-role performance**				**Outcome variable: Extra-role performance**			
	**Coefficients**	**SE**	***t***	**95%CI**	**Coefficients**	**SE**	***t***	**95%CI**
**Independent**								
Burnout	−0.0715	0.0852	−0.8389	−0.2397,0.0968	0.201	0.0312	4.591	0.161,−0.243
**Mediator**								
WD	−0.1627	0.093	−1.74	−0.0837,0.219	−0.243	0.024	5.231	−0.317, −0.218
**Interactive Effect**								
BO × FI	−0.0310	0.1054	−0.294	−0.239,0.177	−0.014	0.0033	−3.2363	−0.0165,−0.0075

**Model Summary**	***R***	***R*^2^**	**MSE**	***F***	***R***	***R*^2^**	**MSE**	***F***

	0.1615	0.0261	0.4908	2.128	0.681	0.463	30.1	386

BO, burnout; WD, work disengagement; FI, Financial insecurity.N = 162. *P < 0.01, and ***p < 0.001.

**TABLE 6:** Index of moderated mediation.

**Moderator: FI**	**Index**	**SE (Boot)**	**Bias corrected bootstrapping 96%**	
			**Lower**	**Upper**
BO > WD > IRP	0.0051	0.236	–0.247	0.0738
BO > WD > ERP	–0.0041	0.0013	–0.0061	–0.0021

*BO, burnout; WD, work disengagement; IRP, In-role performance; ERP, Extra-role performance.*

This study conducted a spotlight analysis to investigate the conditional indirect effect of the moderator on extra-role performance. Accordingly, if the existence of moderated mediation is supported by its index, investigations must be carried out on the indirect effect at representative values of the moderator (shown as conditional indirect effect) (Spiller et al., 2013). This method allows for further exploration of the conditions in which mediation is present or absent (Preacher et al., 2007). Table 7 does not show any significant indirect effect for disengagement with low financial insecurity (effect: 0.0281; 95% CI: −0.1124 to 0.0071). On the other hand, there was a significant effect for both moderate (effect: 0.141; 95% CI: 0.112 to 0.172) and high financial insecurity (effect: 0.015; 95% CI: 0.071 to 0.130). Therefore, it is concluded that burnout affects extra-role performance via work disengagement and the mediation relationship which increases with the increment of financial insecurity. Overall, these results fail to reject H_5_.

**TABLE 7:** Conditional Indirect Effect.

		**In-role performance**				**Extra-role performance**			
**Mediator**		**Effect**	**BootSE**	**Boot LLCI**	**BootULCI**	**Effect**	**BootSE**	**Boot LLCI**	**Boot ULCI**
WD	(−1 SD Fin)	–0.0253	0.0319	0.146	0.228	0.0281	0.022	–0.1124	0.0071
WD	(Mean Fin)	0.0215	0.0216	–0.0762	0.0057	0.141	0.014	0.112	0.172
WD	(+1 SD Fin)	0.0165	0.0267	–0.0721	0.0390	0.208	0.015	0.071	0.130

*WD, work disengagement.*

## Discussion

This present study set out to determine the spillover effects of personal financial insecurity that can impact burnout-disengagement relationships during the COVID-19 pandemic. To do this, we examined whether pandemic-perceived financial insecurity moderated the relationship between burnout and disengagement. Secondly, we assessed the interaction effects of financial insecurity and burnout on the performance of moonlighting employees through work-disengagement. Our results indicate that perceptions of financial insecurity shaped by the COVID-19 crisis had positive effects on work disengagement. The results support existing findings (Wittmer and Martin, 2013), suggesting that moonlighters’ higher perception of financial insecurity leads to reduced work engagement that leaves them with a negative mentality (H_1_ was failed to reject). This result also underscores the negative effect of job demands, as the JD-R theory postulated (Bakker and Demerouti, 2017). Moonlighters who are employed during the COVID-19 crisis tend to experience the dual insecurities of job and finance. When they perceive a stability shortfall in their employer-employee exchange relationship, they respond by withdrawing and disengaging from work (Parzefall and Hakanen, 2010). Compounded by elevated anger, frustration, and negative effects, employees’ capacity to sustain a positive motivational-affective state is held back (Sora et al., 2010).

Another notable finding is the great influence of burnout (during the COVID-19 pandemic) on moonlight work engagement, which is in good agreement with earlier literature (e.g., Bakker et al., 2008). These results indicated that moonlighters who have high levels of burnout experience heightened disengagement of work (H_2_ was failed to reject). Building upon JD-R theory (Bakker and Demerouti, 2017), the present study demonstrated that variations exist across and within organizations in terms of how COVID-19 has impacted job demands and resources. Our results suggest that most employees face deteriorated working conditions, especially among moonlighters. In light of these constraints, COVID-19 has substantially exposed employees to a greater risk of job burnouts which are associated with disengagement from work as well.

The results of the interaction-moderation analysis revealed that financial insecurity moderates the effect of moonlighters’ burnout on their level of disengagement. We found, in line with H_3_, that heightened financial insecurity strengthens the burnout-disengagement relationship, which is a striking contrast when compared to the incidence of low financial insecurity. The results support existing findings that found multiple job-holders’ susceptibility to crushing concerns about their financial situation, exhaustion, burnout, and overall disengagement (Sliter and Boyd, 2014; Bouwhuis et al., 2018). Similar observations were made on the nature of an economic crisis, which plays the role of a macro stressor that consolidates different economic stressors of employees, namely, financial distress and job instability or loss, all of which could trigger the onset of an extended state of stress that eventually leads to psychological distress and burnout (Caraballo-Arias et al., 2018; Giorgi et al., 2020; Sigursteinsdottir et al., 2020). Following the conservation of resources (COR) theory that predicts loss spirals (Hobfoll, 2011), an employee’s exhaustion may deplete resources, thereafter disengaging further in work (Toppinen-Tanner et al., 2002; Diestel and Schmidt, 2010; Tauhed et al., 2019). Prior research also found that job insecurity and financial concerns are associated with suboptimal mental health, specifically impacted by national or global events, such as the COVID-19 crisis (Wilson et al., 2020).

Our study also demonstrated that burnout symptoms that emerged during the COVID-19 crisis depreciate extra-role performance through the work disengagement mediator, conditional on financial insecurity (a boundary condition) for increased work disengagement. The present study provides empirical evidence on the direct link between burnout and disengagement of work, which is similar to previous findings (Tan et al., 2020). Our study confirmed the significance of this direct relationship in congruence with the conditional on an elevated level of financial insecurity. It can be said that the interaction effects of financial insecurity and burnout bring a negative effect on moonlighters’ extra-role performance (H_5_ was failed to reject). Our findings indicated that the lack of resources as a consequence of unforeseen events, coupled with a lack of days off, would inevitably increase burnout and fatigue. The impact of this demand would reduce the employees’ commitment to the organization, increase disengagement, and reduce extra-role performance.

Conversely, there was no major association, directly or indirectly, between disengagement and in-role performance (H_4_ was rejected). Among the plausible explanations for this finding is that it represents the behavioral nature of both types of performances. In the pandemic-induced financial crisis, moonlighters who are financially stacked against and have unfavorable relations with their organization tend to decide that reducing their regular work tasks is not a viable option because as their organization undergoes austerity, particular work behaviors are noticed. Therefore, moonlighters in this environment avoid standing out in a negative light.

The present study makes several notable contributions to the management and organization literature with theoretical and practical implications. We conducted a timely and appropriate examination of moonlighters’ perceived financial insecurity set against the backdrop of the COVID-19 crisis. We also confirmed the organic and causal relationships between financial insecurity, burnout, work disengagement, and in-role and extra-role performances. This establishes a theoretical foundation that explains the connection between financial insecurity and the psychological responses of moonlighting employees. Our present research may be one of the earliest empirical works conducted to verify the significant negative effects of financial insecurity to shed light on how moonlighters are responding and behaving in this time of crisis. Besides, as a matter of interest among organizational management practitioners, our study provides insights into the burnout-financial insecurity interactions that affect employees’ level of disengagement and performance at the workplace. The academic opportunity for theoretical development is presented - one that explores justifications on placating financial insecurity. More importantly, we highlight practical implications for human resource practitioners. It appears that the COVID-19 crisis is far from gaining a quick resolution. The results indicate that if organizations withhold long term assistance or incentive programs to establish financial security for moonlighters, during and after the crisis, its unfavorable side effects may spill over and influence other organizational members by nature of a multipronged attack, thus making it much harder for managerial interventions. Concurrently, there is an urgent need to advance understanding of organizational support mechanisms that reduce disengagement and boost engagement among moonlighters. They need security blanket resources to face pandemic-specific and uncertain job demands. In response, organizations can intervene through top-down or bottom-up approaches or mechanisms to ensure employee wellness, as a means to restore the JD-R equilibrium.

The most obvious finding to emerge from our study is that the dual insecurities of job and finance lay a path laden with worrying consequences on employee mental health. It makes sense for the organization to pay attention to employees who are experiencing depressive symptoms during this crisis. Importance needs to be placed, on the part of employers, in exercising mindfulness in placating employees’ feelings of uncertainty, by being the source of hope. To respond to employees who are enduring burnout symptoms, employer intervention could be carried out to address financial concerns. As an option, employers can promote telecommuting, with or without reduced hours and income, to ensure that some form of income, although not in its entirety, can be safeguarded. This is in line with previous findings that demonstrated job resources playing a weaker role as compared with job demands in predicting job stress. This suggests that demand-prompted policies may be more effective in reducing stressors. Nevertheless, improving job resources should not be discounted. Nurturing and supportive relationships at the workplace is a valuable resource, and if tended carefully, it sets off a “virtuous cycle” that functions as a job-stress reliever. According to Hovey et al. (2014), relationships are the foundation of positive psychology in which the mechanism of emotional and social support is established, thus facilitating continued human evolution and bonding. These relationships provide and strengthen self-efficacy to overcome times of hardship, such as pandemic-prompted financial and economic crisis. It was expected that the psychological benefits of social support mitigated the perception of financial threat concerning life satisfaction in the group of individuals with higher levels of emotional support, in detriment of the group with lower levels of emotional support.

Interestingly, moonlighters who are severely financially impacted are more likely to prevail when social support avails as a coping mechanism as compared with those with less social support access (Åslund et al., 2014). As our results indicate that emotional discord runs parallel with burnout which reduces in-role performance, it is of the interest of the management, therefore, to consider ways to placate emotional discord. Therefore, what is now needed is a study on how HR moonlighting policies affect outcomes for employers and employees alike.

This study acknowledges several strengths and limitations in the aspects of research design. We acknowledged results generalizability issues due to the small sample size. To address this, we adopted the quantitative method and the participant selection process to arrive at an improved generalization of the findings. Quantitative methods produce factual, reliable results that can be generalizable to a larger population (Nardi, 2018). Besides, to generalize the results of the study to similar public and private employees who moonlight, we used a systematic sampling method where we selected members of the population at a regular interval, and we included criteria for participant selection. Despite the limitations, this study provides some insights for future research operationalization. First, our cross-sectional analysis did not yield a conclusion on the causal relationship. Having said that, the experimental research design is better matched to describe a uniform causal direction of the relationships among the constructs. Second, a longitudinal research design allows further assessment of COVID-19’s post and long-term effects on employees. Although we tried to examine financial insecurity during the pandemic and its potential impact on moonlighters’ burnout, disengagement, and performance, our study might have failed to consider and/or observe all confounding factors. Future research should scrutinize additional factors that might also be influenced by financial insecurity, such as the relationship between moonlighters’ adaptation styles or personality and performance. Finally, what is needed is to further assess the effect of the COVID-19 crisis on employee performance as this is compounded on existing occupational stress across all sectors.

## Conclusion

The findings of the present study point out that financial insecurity, during the pandemic, spills over the exchange relationship of burnout and work disengagement, thus implicating interferences in moonlighters’ performance in the organization. Our research explored whether financial insecurity moderates the effect of burnout on disengagement, and how this disengagement acts as a mediating factor in this process. Responding to the critical need, we identified the conditions and mechanisms in which burnout may cause effect variations in performance outcomes. The results of this study exposed financial insecurity as the conditional factor that interacts with the mediation outcome of disengagement between the relationship of burnout and extra-role performance. Explicitly, when the condition of high financial insecurity occurs, the results showed the mechanism of disengagement outcome is stronger between the relationships of burnout and extra-role performance. In this regard, burnout predicted extra-role performance through a moderated (financial insecurity) and mediation (work disengagement) relationship. In conclusion, this study deduces that the provision of immediate resources to moonlighters could alleviate short-term financial instability, therein minimizing their burnout and disengagement levels at work.

## Data Availability Statement

The raw data supporting the conclusion of this article will be made available by the authors, without undue reservation.

## Ethics Statement

UPM Ethics Committee for Research Involving Human Subject approved the study protocol as registered as JKEUPM-2020-180. The patients/participants provided their written informed consent to participate in this study.

## Author Contributions

RR and ZZ made substantial contributions to the conception of the work, the experimental design, the acquisition of data, the analysis and interpretation of data, and drafting the manuscript and agreed to be accountable for all aspects of the work in ensuring that questions related to the accuracy or integrity of any part of the work are appropriately investigated and resolved. ZZ made substantial contributions to the analysis and interpretation of data and revising the manuscript critically for important intellectual content. SA made substantial contributions to the analysis of data and revising the manuscript critically for important intellectual content. All authors contributed to the article and approved the submitted version.

## Conflict of Interest

The authors declare that the research was conducted in the absence of any commercial or financial relationships that could be construed as a potential conflict of interest.
